# An Ultrasensitive Gold Nanoparticle-based Lateral Flow Test for the Detection of Active Botulinum Neurotoxin Type A

**DOI:** 10.1186/s11671-017-1944-9

**Published:** 2017-03-29

**Authors:** Jing Liu, Shan Gao, Lin Kang, Bin Ji, Wenwen Xin, Jingjing Kang, Ping Li, Jie Gao, Hanbin Wang, Jinglin Wang, Hao Yang

**Affiliations:** 1 State Key Laboratory of Pathogen and Biosecurity, Institute of Microbiology and Epidemiology, Beijing, 100071 People’s Republic of China; 2 The 307th Hospital of Military Chinese People’s Liberation Army, Beijing, 100071 People’s Republic of China

**Keywords:** Botulism, Botulinum toxin, Lateral flow assay, Endopeptidase activity assay, Mouse lethality assay

## Abstract

Botulism is a severe and potentially lethal paralytic disease caused by several botulinum neurotoxin-producing Clostridia spp. In China, the majority of the cases caused by botulism were from less-developed rural areas. Here, we designed specific substrate peptides and reconfigured gold nanoparticle-based lateral flow test strip (LFTS) to develop an endopeptidase-based lateral flow assay for the diagnosis of botulism. We performed this lateral flow assay on botulinum neurotoxin-spiked human serum samples. The as-prepared LFTS had excellent performance in the detection of botulinum neurotoxin using only 1 μL of simulated serum, and its sensitivity and specificity were comparable to that of mouse lethality assay. Moreover, the assay takes only half a day and does not require highly trained laboratory staff, specialized facility, or equipment. Finally, our LFTS can be potentially extended to other serotypes of BoNTs by designing specific substrate peptides against the different types of BoNTs. Overall, we demonstrate a strategy by which LFTS and endopeptidase activity assays can be integrated to achieve facile and economic diagnosis of botulism in resource-limited settings.

## Background

The botulinum neurotoxins (BoNT) are produced by several Clostridia spp. as single 150-kDa polypeptide chains, which consist of a 100-kDa heavy chain joined by a disulfide bond to a 50-kDa light chain. Serotypically, this deadly toxin includes seven distinct types from BoNT/A to BoNT/G and possesses the metallopeptidase activity specific for the soluble *N*-ethylmaleimide-sensitive factor activating protein receptor (SNARE) complex. It could block the neurotransmitter release of the intoxicated peripheral neurons and cause death in consequence of blockade of respiratory function in vertebrates [1–4]. Among the seven serotypes, BoNT/A and B are commonly associated with human botulism, but the former causes more severe consequences than the latter [5].

Due to the severity of botulism, along with clinical manifestion, early detection of BoNTs in patients is required to deliver appropriate treatment. Currently, the detection of BoNTs is mainly based on biological and immunological techniques following culturing of suspicious strains. For decades, mouse lethality assay (MLA) has still served as the “gold standard method” approved by the Association of Official Analytical Chemists (AOAC) for the analysis of BoNTs, which is able to detect approximately 10 pg/mL of the toxin [6–9]. However, MLA is costly (requirements of expensive animal facilities) and time consuming (up to 4 days), and most important, it involves in the ethical problems for the use of live animals [9, 10]. The most frequently used immunological method for toxin detection and serotyping is sandwich-based enzyme-linked immunosorbent assay (ELISA). Its ease of use, good specificity, and high-throughput capabilities make it suitable for the application in routine laboratories [11]. Nevertheless, the major drawback is that it lacks capacity to differentiate active BoNTs from the inactive toxins. Moreover, its application in resource-limited areas is restricted owing to the requirement for specialized microplate reader and tedious procedure.

**Fig. 1:**
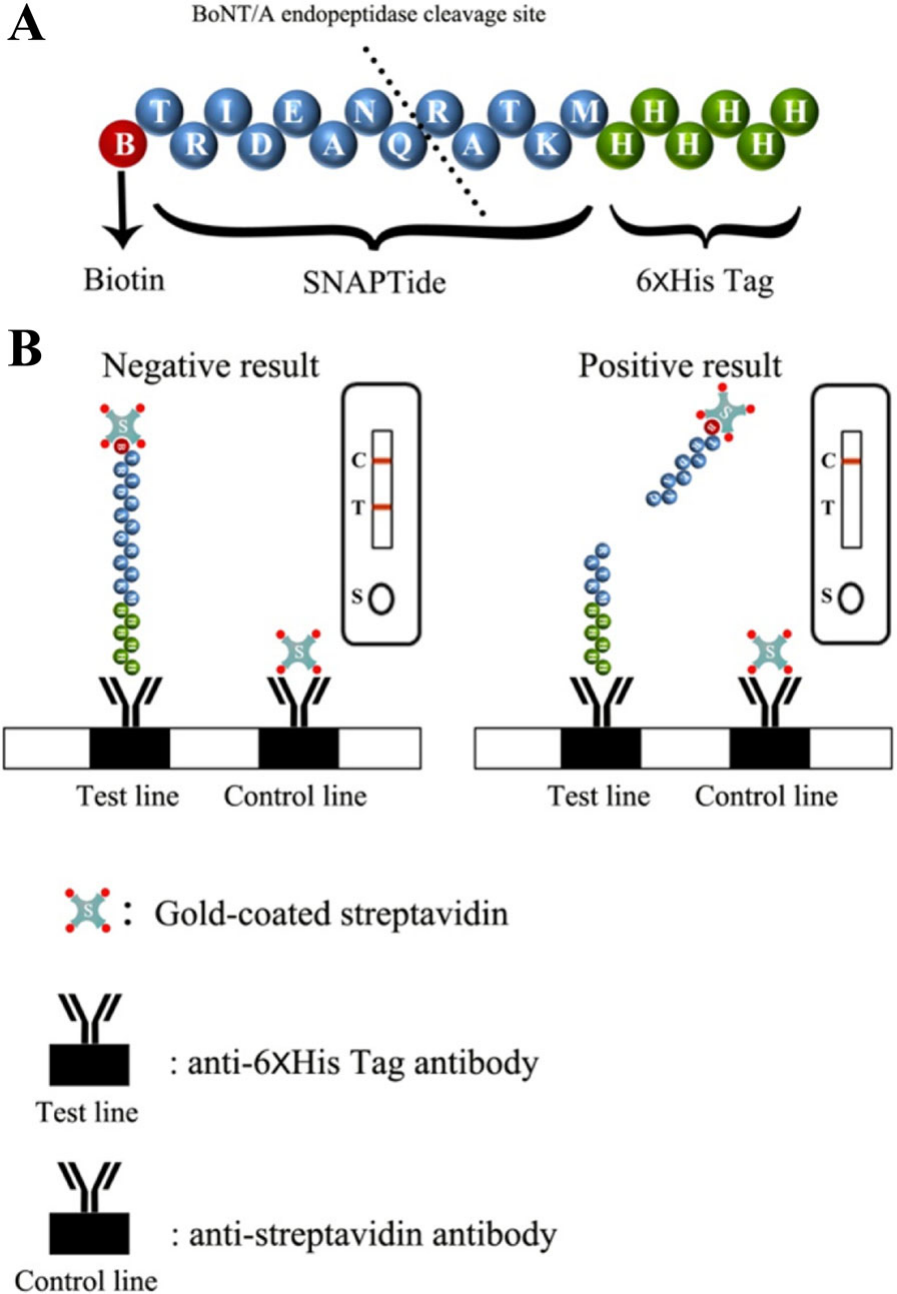
The diagrammatic representation of the as-prepared LFTS. **a** The sequence of specific substrate peptide and its cleavage site. **b** The schematic principle of the LFTS based on the endopeptidase activity of BoNT/A (The *inset* is the diagrammatic drawing of test results, *C*: control line; *T*: test line; *S*: sample pad)

In this article, we developed a very easy gold nanoparticle-based LFTS (lateral flow test strip) to detect active BoNT/A in patients’ serum sample. It is well known that BoNT/A selectively cleaves one of the three SNARE polypeptides, SNAP-25 (25-kDa synaptosome associated protein). Therefore, the zinc-dependent endopeptidase activity of BoNT/A was utilized to design a specific substrate peptide with a His-tag at the carboxyl terminal end and a biotin at the amino terminus (Fig. 1a). The sequence of specific cleavage site was based on SNAPtide®, a very short SNAP-25 homologue with a unique cleavage site for BoNT/A [12, 13]. After incubation with BoNT/A-spiked serum sample for a certain time duration, the biotin at the amino terminus of the peptide was cleaved off, resulting in that gold nanoparticle-labeled streptavidin-substrate peptide complexes could not be captured on the test zone of LFTS, thus, LFTS presented a different coloration from BoNT/A-negative result (Fig. 1b).

## Results

### Purification and Characterization of BoNTs

**Fig. 2:**
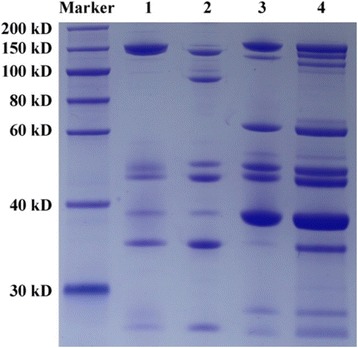
The SDS–PAGE analysis of the purified BoNT/A and BoNT/B complex. (*Lane 1*: BoNT/A; *Lane 2*: DTT-treated BoNT/A; *Lane 3*: BoNT/B; *Lane 4*: DTT-treated BoNT/B)

Since the differences in purification methods or manufacturing processes may influence the activity of BoNTs, there remains a paucity of appropriate and comparable quantitative data of BoNTs analysis from variety of methods based on its metallopeptidase activity [14, 15]. In order to provide an objective information for the comparison between various analytic methods of BoNTs, we quantified the as-prepared BoNTs by BCA protein assay and determined its lethal toxicity by MLA. The results of SDS-PAGE showed that the purified BoNTs were the complexes with hemagglutinin (HA) and non-toxic non-HA (NTNH) (Fig. 2). After the treatment of dithiothreitol (DTT), the interchain disulfide bond in both serotypes could be reduced resulting in 100 kD heavy chain and 50 kDa light chain (Fig. 2, lane 2 and 4). Because the BoNT/B was the crude extract by DEAE-sepharose chromatography in this work, its purity was clearly below that of BoNT/A (Fig. 2, lane 3 and 4). The results of BCA protein assay showed that the concentrations of the BoNT/A and BoNT/B were 0.2 mg/mL and 11.3 mg/mL respectively. The data of MLA indicated that the lethal toxicity were 1.3 × 10^7^ median lethal dose (LD_50_)/mL for BoNT/A and 1 × 10^6^ LD_50_/mL for BoNT/B (Table. 1).

**Table 1:** The lethal toxicity of BoNT/A and B calculated by Reed–Muench method [29]

	Dilution	Died	Survived	Cumulative total		
				Died	Survived	Percent mortality (%)
BoNT/A	1:5 × 10^6^	3	1	3	1	75
	1:1 × 10^7^	0	4	0	5	0
	1:2 × 10^7^	0	4	0	9	0
BoNT/B	1:2 × 10^5^	4	0	7	0	100
	1:4 × 10^5^	3	1	3	1	75
	1:8 × 10^5^	0	4	0	5	0

### The Optimization and Performance of LFTS

**Fig. 3:**
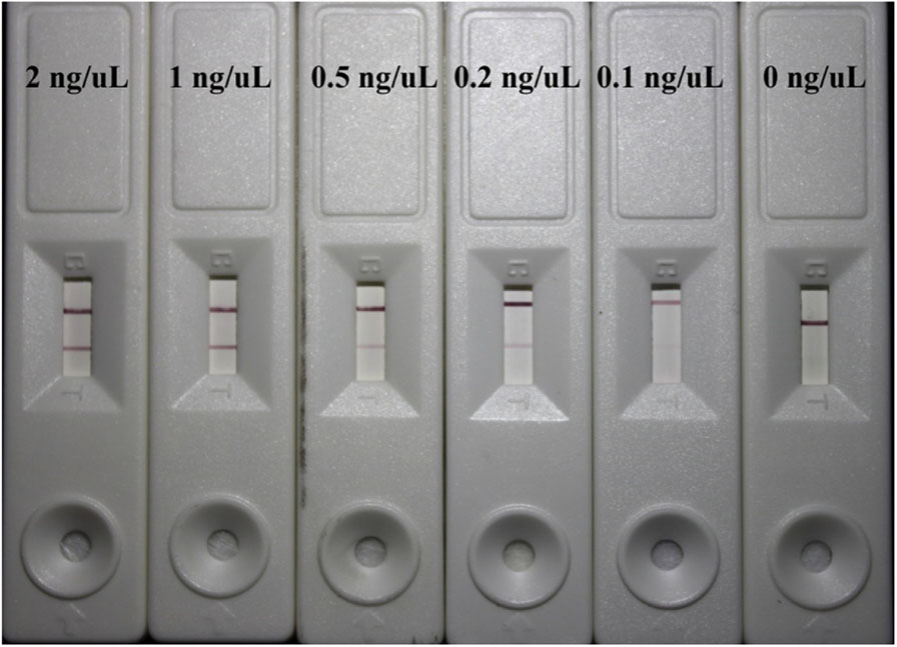
The determination of the minimum quantity of the substrate peptide. The concentrations of substrate peptide used in the experiments were from 0 to 2 ng/μL (*from right to left*)

Theoretically, the intensity of the color depicted in test lines was determined by the quantity of the substrate peptide when the amount of gold nanoparticle conjugates is constant. However, the excess quantity of the substrate peptide would inevitably result in prolongation of the enzymolysis time thus reducing the sensitivity of the assay within the given digestion time. Therefore, the substrate peptide was diluted serially and applied into the LFTS to determine the minimum quantity of substrate peptide which could clearly generate a red visual signal in test line. In this work, the minimum quantity of the substrate peptide was found to be 0.2 ng/μL, but the intensity of the color was visibly less clear than that of 1 ng/μL. As a result, 1 ng/μL was adopted as the optimized concentration of the substrate peptide for the subsequent test (Fig. 3).

**Fig. 4:**
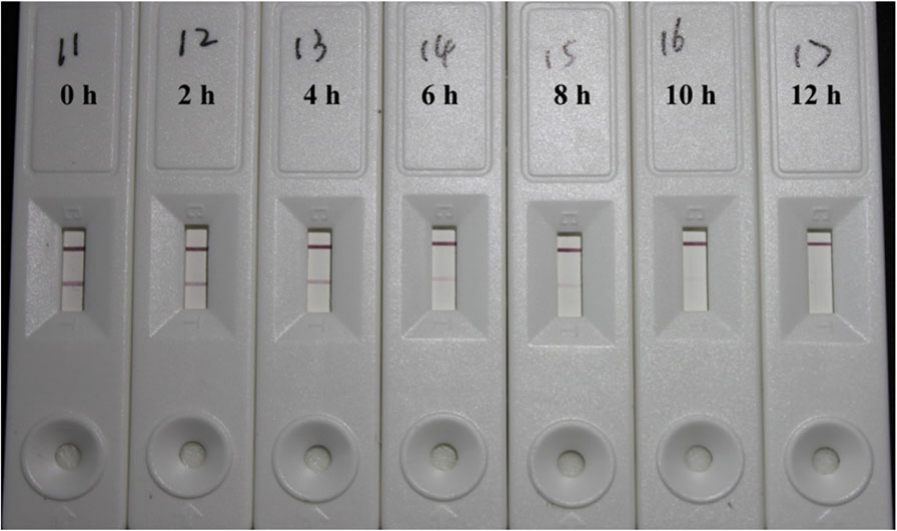
The determination of the optimal digestion time of the LFTS. The digestion time were from 0 to 12 h (*from left to right*)

Because the limit of detection (LOD) of MLA for the purified BoNT/A was approximately 20 pg/mL, this LOD was artificially taken as the goal that our LFTS should achieve and used to explore the optimal digestion time. According to the results, the LFTS generated significant difference in colorization after 6 h of digestion of the substrate peptide by 20 pg/mL BoNT/A-spiked PBS solution and the red signal in test line disappeared completely after 12 h of incubation (Fig. 4). As a consequence, 12 h was determined as the optimal digestion time in the later experiments.

**Fig. 5:**
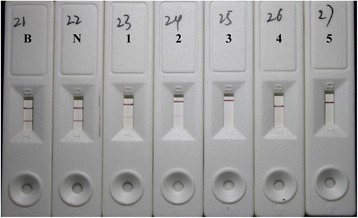
The specifity and sensitivity of the LFTS. (*B*: BoNT-negative serum; *N*: BoNT/B-spiked serum; *1 ~ 5*: 0.2, 2, 20, 200, and 2 ng/mL BoNT/A-spiked sera respectively)

SNAPtide is a synthetic, commercially available, 13-amino acid peptide that contains the native SNAP-25 cleavage site for BoNT/A, the specifity of which has been confirmed extensively [16–18]. Hence, we only used BoNT/B-spiked and BoNT-negative sera to test the specifity of the LFTS in this study. The results of these two samples showed that both LFTSs displayed visible test and control lines, validating the proper specifity of the assay (Fig. 5b and N strips). BoNT-negative sera were spiked with purified BoNT/A to establish the LOD for the LFTS. BoNT/A was serially diluted in sera from 2 to 0.2 pg/mL, and the qualitative detection by visual inspection revealed a significant decrease in test line density with an LOD of 20 pg/mL which was equivalent to 1.3 LD_50_/mL (Fig. 5 No. 1 ~5 strips). Besides, MLA was conducted with the same BoNT/A-spike samples and the LOD were found to be 20 pg/mL which is consistent with that of the LFTS.

## Discussion

Botulism is a severe and potentially lethal paralytic disease characterized by symmetric cranial nerve palsy, commonly followed by symmetric, descending, flaccid paralysis of involuntary muscles, which may result in respiratory compromise and death [19]. The mortality among the confirmed cases of botulism is reported to be 3–10% and that of untreated cases could reach up to 40% or more [20–22]. The key to treat botulism lies in early diagnosis, intensive supportive care, and timely botulinum antitoxin administration. Particularly in suspected infant botulism cases, the human-derived botulinum antitoxin has been recommended to administer as early as possible by some experts [19]. Moreover, unlike the medication for common bacterial infections, antibiotics are not recommended for infant botulism because antibiotic administration may result in *Clostridium botulinum* lysis and subsequent BoNTs release into the gut lumen [19, 23]. Therefore, reducing the time needed for clinical diagnosis plays an absolutely vital role in the treatment for botulism.

As noted before, MLA is universally accepted for confirming BoNT by international regulatory agencies. However, this method takes up to 4 days, or even longer if toxin is to be serotyped. The other drawbacks include the laborious operating procedure, expensive animal care facilities, and health risks by conducting animal experiments and ethical objections. In past decades, significant progress has been made to develop alternative in vitro detection methods such as immunoassays, endopeptidase activity assays, mass spectrometry, cell-based assays, and so on [24]. Many of them have achieved the sensitivities near or below that of MLA. It is worth mentioning that most tests require highly trained laboratory staff, specialized equipment and material. Gold nanopartle-based LFTS are self-contained without the need for sophisticated equipment or an expert analyst [25]. Thus it is ideal for field situations or resource-limited settings. Nevertheless, based on current double-antibody sandwich format, the sensitivity of LFTS is inferior to that of MLA.

Our LFTS assay combines the very best of LFTS and endopeptidase activity assays. The former offers a familiar and easy-to-use laboratory assay format. The latter provides the specificity to BoNT/A and enzyme-based signal amplification processes. Its sensitivity of 20 pg/mL approaches that of MLA (10 pg/mL). Compared with traditional MLA or ELISA methods, the assay takes only half a day and consumes significantly less sample and reagents, which will significantly reduce assay cost and simplify disposal. In China, the majority of the cases caused by botulism were from less-developed rural areas [26]. This assay may contribute to the improvement of treatment for botulism without any requirements for skilled laboratory staff, additional facilities, or equipments. Finally, our LFTS can be potentially extended to other serotypes of BoNTs by designing specific substrate peptides. Overall, we demonstrate a strategy by which LFTS and endopeptidase activity assays can be integrated to achieve on-site diagnosis of botulism in resource-limited settings.﻿

## Conclusions

In this work, we designed specific substrate peptides and reconfigured gold nanoparticle-based lateral flow test strip (LFTS) to develop an endopeptidase-based lateral flow assay for the diagnosis of botulism. The LFTS consumes only 1 μL of serum sample and had excellent sensitivity and specificity which were comparable to those of mouse lethality assay. Moreover, the assay takes only half a day and does not require highly trained laboratory staff, specialized facility, or equipment. Finally, our LFTS can be potentially extended to other serotypes of BoNTs by designing specific substrate peptides against the different types of BoNTs. All in all, we demonstrate a strategy by which LFTS and endopeptidase activity assays can be integrated to achieve facile and economic diagnosis of botulism in resource-limited settings.﻿

## Methods

### Materials

Gold-in-a-Box™ conjugation kit (20 nm, 15 OD gold nanoparticle) was purchased from BioAssay Works, LLC (Ijamsville, MD, USA). HiPrep DEAE FF 16/10 and HiPrep 16/60 Sephacryl S-200 HR were from GE Healthcare Bio-Sciences AB (Uppsala, Sweden). C. botulinum type A strain 62A and C. botulinum type B strain 621 were obtained from the bacterial culture collection of Beijing Institute of Microbiology and Epidemiology. Streptavidin, and anti-Streptavidin antibodies were bought from ProSpec-Tany TechnoGene Ltd (Ness Ziona, Israel), and anti-His monoclonal antibodies was bought from Abbkine Inc (Redlands, CA, USA). Pierce® BCA Protein Assay Kit was purchased from Thermo Fisher Scientific Inc (Waltham, MA, USA). The substrate peptide with a His-tag at the carboxyl terminal end and a biotin at the amino terminus was synthesized by GL Biochem (Shanghai) Ltd (Shanghai, China). High-flow nitrocellulose membranes, glass fiber, and absorption pad were supplied by Millipore (Billerica, MA, USA). All preparations and measurements were carried out in deionized water as solvent with 18 MΩ/cm or less. All other reagents were purchased domestically. BoNT/A and B-negative sera were obtained from volunteer blood donors. Specific pathogen-free (SPF) female BALB/c mice were purchased from Beijing Vital River Laboratory Animal Technology Co. Ltd, Beijing, China. All animal experiments used in this study were performed according to the protocols approved by the Institutional Animal Care and Use Committee of Beijing Institute of Microbiology and Epidemiology (Identification code for mice: IACUC of AMMS-12-2015-012; Date of approval: 9 December 2015).

### Purification of BoNTs

The C. botulinum type A strain was cultured in TPYG medium (3% bacto peptone, 0.5% glucose, 0.5% yeast extract and 0.05% L-cysteine HCl, 0.025% Na-thioglycolic acid,pH 7.5,) for 20 h at 37 °C, then transferred to TPOM (yeast extract 1%, casein hydrolysate 2%, Na-thioglycolic acid 0.05%, L-cysteine HCl 0.1% and glucose 0.5% pH 7.2–7.4) at the ratio of 1:100 and cultured for 4 days at the same temperature. After the toxins were released from the cytosol following bacterial autolysis, 3 N H_2_SO_4_ was dropped into the medium until its pH value reached 3.4. The medium was kept at 4 °C overnight and then centrifuged at 12,000 × g for 30 min. The supernatant was discarded, and the precipitates were dissolved with 0.2 M PBS (pH 6.0) under stirring for 1 h. The solution was centrifuged at 12,000 × g for 30 min. Ribonuclease was added into the supernatant at a final concentration of 100 μg/mL and incubated at 34 °C for 3 h. Thereafter, the crude BoNT/A were precipitated with 60% ammonium sulfate at 4 °C overnight. After centrifugation at 10,000 × g for 30 min, the precipitates are dissolved in 0.05 M citric acid buffer (pH 5.5). After another centrifugation at 10,000 × g for 30 min, BoNT/A in the supernatant was purified by DEAE-Sepharose and Sephacryl-200 column successively. The purification of BoNT/B was completed according to the previous methods published by our laboratory without polyethylene glycol (PEG) precipitation and Sephacryl-200 column process [27]. BCA (bicinchoninic acid) protein assay and SDS-PAGE were used to analyze the two toxins.

### Lethal Toxicity of the As-Prepared BoNTs

The lethal toxicity of the as-prepared BoNTs was determined according to the previous method [28]. Briefly, the BoNTs were diluted twofold serially in 0.02% gelatin-0.05 M acetate buffer, pH 6.0 from 5 × 10^6^ to 2 × 10^7^ for BoNT/A and from 2 × 10^5^ to 8 × 10^5^ for BoNT/B. Four mice were injected with 0.5 mL doses of each dilution peritoneally and observed for 4 days. The LD_50_ was calculated by the Reed–Muench method [29].

### Preparation of Lateral Flow Test Strips

The anti-His monoclonal antibodies and the anti-streptavidin antibody were dispensed on the nitrocellulose membrane with Biodot XYZ3000 dispenser at concentration of 1 mg/mL as test line and control line respectively. The nitrocellulose membrane was then dried for 30 min. The colloidal gold conjugates were prepared according to the producer’s instruction and printed on conjugate pad at the proportion of 4 uL per test strip and dried at room temperature. Absorbent pad, nitrocellulose membrane, conjugate pad, and sample pad were pasted on the laminating card in order with proper overlapping distance. Eventually, the whole card was cut into final test strips of 4 mm in width with guillotine cutter.

### Optimization of Detection Conditions

The substrate peptide was first dissolved in 0.01 M PBST containing 0.5 mM ZnCl_2_ (pH 6.0, 0.05% Tween-20) at the concentration of 0.1, 0.2, 0.5, 1, and 2 ng/μL. One microliter of substrate peptides mentioned above were blended with 100 μL PBST respectively and then added into the sample pad of LFTS. Five minutes later, the colorization on test zone was used to determine the minimum quantity of the substrate peptide used in digestion step. In addition, 1 μL BoNT/A (20 pg/mL) and 1 μL substrate peptide solution were mixed thoroughly in 18 μL 0.01 M PBST containing 0.5 mM ZnCl_2_ and incubated at 37 °C for 0, 2, 4, 6, 8, 10, and 12 h respectively. The substrate peptide solution digested for different durations was then blended with 100 μL PBST and added into the sample pad of LFTS. Five minutes later, the colorization on test zone was used to determine the minimum digestion time.

### Detection of BoNT/A-Spiked Samples

Firstly, the collected BoNT-negative sera were kept in room temperature for 10 min before use. Then, the purified BoNT/A was spiked in the sera at the concentrations from 2 ng/mL to 0.2 pg/mL to simulate clinical samples. The purified BoNT/B-spiked and BoNT-negative sera were used as negative and blank samples respectively. To determine the LOD of this strategy, 1 μL serum samples spiked with different concentrations of BoNT/A and 1 μL substrate peptide solution were added into 18 μL 0.01 M PBST containing 0.5 mM ZnCl_2_ and incubated at 37 °C for the optimized digestion duration. Afterwards, LFTSs were applied to those samples by following the process previously mentioned again. Meanwhile, MLA was used as the “gold standard method” for methodological comparison.

## Abbreviations

AOAC: Association of Official Analytical Chemists
BCA: Bicinchoninic acid
BoNT: Botulinum neurotoxin
BoNT/A: Botulinum neurotoxin type A
BoNT/B: Botulinum neurotoxin type B
ELISA: Enzyme-linked immunosorbent assay
HA: Hemagglutinin
LD_50_: Median lethal dose
LFTS: Lateral flow test strip
LOD: Limit of detection
MLA: Mouse lethality assay
NTNH: Non-toxic non-HA
PBST: Phosphate Buffer Solution with Tween
PEG: Polyethylene glycol
SNAP-25: 25-kDa synaptosome associated protein
SNARE: Soluble N-ethylmaleimide-sensitive factor activating protein receptor
SPF: Specific pathogen-free

## References

[1] Gao YL, Gao S, Kang L, Nie C, Wang JL (2010). Expression of Hc fragment from Clostridium botulinum neurotoxin serotype B in Escherichia coli and its use as a good immunogen. Hum Vaccin.

[2] Rossetto O, Megighian A, Scorzeto M, Montecucco C (2013). Botulinum neurotoxins. Toxicon.

[3] Simpson L (2013). The life history of a botulinum toxin molecule. Toxicon.

[4] You Z, Yang H, Xin W, Kang L, Gao S, Wang J, Zhang T (2014). Preparation of egg yolk antibodies against BoNT/B and their passive protection in mouse models. Hum Vaccin Immunother.

[5] Rossetto O, Pirazzini M, Montecucco C (2014). Botulinum neurotoxins: genetic, structural and mechanistic insights. Nat Rev Microbiol.

[6] Bigalke H, Rummel A (2015). Botulinum neurotoxins: qualitative and quantitative analysis using the mouse phrenic nerve hemidiaphragm assay (MPN). Toxins.

[7] Dorner MB, Schulz KM, Kull S, Dorner BG (2013). Complexity of botulinum neurotoxins: challenges for detection technology. Curr Top Microbiol Immunol.

[8] Grenda T, Kukier E, Kwiatek K (2014). Methods and difficulties in detection of Clostridium botulinum and its toxins. Pol J Vet Sci.

[9] Halliwell J, Gwenin C (2013). A label free colorimetric assay for the detection of active botulinum neurotoxin type A by SNAP-25 conjugated colloidal gold. Toxins.

[10] Rasooly R, Do PM (2008). Development of an in vitro activity assay as an alternative to the mouse bioassay for Clostridium botulinum neurotoxin type A. Appl Environ Microbiol.

[11] Stanker LH, Merrill P, Scotcher MC, Cheng LW (2008). Development and partial characterization of high-affinity monoclonal antibodies for botulinum toxin type A and their use in analysis of milk by sandwich ELISA. J Immunol Methods.

[12] Baldwin MR, Bradshaw M, Johnson EA, Barbieri JT (2004). The C-terminus of botulinum neurotoxin type A light chain contributes to solubility, catalysis, and stability. Protein Expr Purif.

[13] Feltrup TM, Singh BR (2012). Development of a fluorescence internal quenching correction factor to correct botulinum neurotoxin type A endopeptidase kinetics using SNAPtide. Anal Chem.

[14] Gart MS, Gutowski KA (2015). Aesthetic uses of neuromodulators: current uses and future directions. Plast Reconstr Surg.

[15] Wilson AJ, Chang B, Taglienti AJ, Chin BC, Chang CS, Folsom N, Percec I (2016). A quantitative analysis of OnabotulinumtoxinA, AbobotulinumtoxinA, and IncobotulinumtoxinA: a randomized, double-blind, prospective clinical trial of comparative dynamic strain reduction. Plast Reconstr Surg.

[16] Bagramyan K, Barash JR, Arnon SS, Kalkum M (2008). Attomolar detection of botulinum toxin type A in complex biological matrices. PLoS One.

[17] Joshi SG (2012). Detection of biologically active botulinum neurotoxin—A in serum using high-throughput FRET-assay. J Pharmacol Toxicol Methods.

[18] Ouimet T, Duquesnoy S, Poras H, Fournie-Zaluski MC, Roques BP (2013). Comparison of fluorigenic peptide substrates PL50, SNAPTide, and BoTest A/E for BoNT/A detection and quantification: exosite binding confers high-assay sensitivity. J Biomol Screen.

[19] Carrillo-Marquez MA (2016). Botulism. Pediatr Rev.

[20] Chalk CH, Benstead TJ, Keezer M (2014). Medical treatment for botulism. Cochrane Database Syst Rev.

[21] Feng L, Chen X, Liu S, Zhou Z, Yang R (2015). Two-family outbreak of botulism associated with the consumption of smoked ribs in Sichuan Province, China. Int J Infect Dis.

[22] Proverbio MR, Lamba M, Rossi A, Siani P (2016). Early diagnosis and treatment in a child with foodborne botulism. Anaerobe.

[23] Zhang J, Xu W, Zhao M, Wu Y, Zhang X, Zhang C, Wang Y, Liu X, Lu S, Xu X (2016). Clinical analysis of three cases with infant botulism and review of literature. Chin J Pediatr.

[24] Singh AK, Stanker LH, Sharma SK (2013). Botulinum neurotoxin: where are we with detection technologies?. Crit Rev Microbiol.

[25] Wang K, Qin W, Hou Y, Xiao K (2016). The application of lateral flow immunoassay in point of care testing: a review. Nano Biomed Eng.

[26] Zhang S, Wang Y, Qiu S, Dong Y, Xu Y, Jiang D, Fu X, Zhang J, He J, Jia L (2010). Multilocus outbreak of foodborne botulism linked to contaminated sausage in Hebei province, China. Clin Infect Dis.

[27] Zhao Y, Kang L, Gao S, Gao X, Xin W, Wang J (2012) PEG precipitation coupled with chromatography is a new and sufficient method for the purification of botulinum neurotoxin type B. PLoS One 7(6):e39670 10.1371/journal.pone.0039670 PMC3386254 22761863

[28] Kondo H, Shimizu T, Kubonoya M, Izumi N, Takahashi M, Sakaguchi G (1984). Titration of botulinum toxins for lethal toxicity by intravenous injection into mice. Jpn J Med Sci Biol.

[29] Reed LJ, Muench H (1938). A simple method of estimating fifty percent endpoints. Am J Epidemiol.

